# “A Comparative Analysis of the Number of Organ Retrievals in Relation to Potential Donation Qualifications in Populations of Patients From a Single Anesthesiology and Intensive Care Unit in 2017-2018”

**DOI:** 10.3389/fmed.2022.930290

**Published:** 2022-08-17

**Authors:** Bartosz Kudliński, Marta Trosiak, Magdalena Wojciech, Gabriel Zaborniak, Jagoda Kania, Aleksandra Buczek, Olga Fedorowicz, Marek Myślak

**Affiliations:** ^1^Department of Emergency Medicine, Anesthesiology and Intensive Care in University Hospital Named After K. Marcinkowski, Collegium Medicum University of Zielona Gora, Zielona Góra, Poland; ^2^Department of Anesthesiology and Intensive Care, University Hospital Named After K. Marcinkowski, Zielona Góra, Poland; ^3^Department of Statisical Mathematics and Econometry, Faculty of Mathematics, Informatics and Econometry in University of Zielona Gora, Zielona Góra, Poland; ^4^Faculty of Mathematics, Informatics and Econometry in University of Zielona Gora, Zielona Góra, Poland; ^5^Faculty of Medicine and Health Science, Collegium Medicum University of Zielona Gora, Zielona Góra, Poland; ^6^Department of Nephrology, Transplantology and Internal Medicine Clinic, Pomerian Medical University, Szczecin, Poland

**Keywords:** brain death, organ donation, organ procurements, transplantation, brain trauma, cerebral function monitoring, CT-angiogram

## Abstract

The population of patients declared as brain dead and qualified for organ donation is relatively low in Poland. The main causes of brain death include cerebral vascular diseases and brain trauma (54 and 34%, respectively, according to Poltransplant registry data). The number of organ procurements in Poland is constantly recorded on average at 14 donations per 1 million citizens (14/mln) in 2017 and 12 donations per one million in 2018. It is difficult to precisely define the number of patients who meet the criteria for brain death certification. The authors have retrospectively analyzed the medical data of 229 patients from 2017 and 2018 records with the aim of identifying potential organ donors among patients of the Intensive Care Unit (ICU) in the University Hospital in Western Poland. Brain death was suspected in 53 patients (23.14%). Brain imaging to confirm no cerebral flow (which is consistent with brain death) was performed in 17 patients (7.45%) and this, as a result, led to organ donation in 9 cases (3.93%). The factors identified as having a positive influence on organ donation included: daily thorough physical examination, (Glasgow Coma Scale) GCS assessment, depth and duration of sedation, ICU length of stay and early performance of a CT-angiogram.

## Introduction

Polish transplantolgy faces the same problem which is a worldwide struggle. The number of patients waiting on transplant lists is rising and the average donation number per year remains unchanged. This, as a result, leads to prolonged wait time for transplant patients, not to mention the wait-list mortality and increased mortality following delayed deceased transplantation (1). Polish law allows organ retrieval if the donor didn't object in the lifetime and isn't included in Central Register of Objections. The approval of the donor's family is not obligatory. The average number of organ procurements in Poland is constant, at the level of 13 donations per 1 million citizens, which ranks us far behind the leaders in Europe. The authors have retrospectively analyzed the number of organ procurements in their hospital with a view to potential improvement in the process of potential donors identification.

According to the data published by Poltransplant in July 2019 (2) there were 388 hospitals with “donation potential” registered in Poland which means that they fulfill every requirement to declare brain death and provide essential care of the donor. The number includes a persistent group of hospitals actively engaged in a donation program during the years 2013–2018, however, the number decreased from 142 hospitals in 2013 to 138 hospitals in 2018.

Table 1 shows that an average of 34.9% of hospitals with donation potential were active during qualification to donation and organ retrieval. The average number of donors was 13 per one million of province inhabitants. What is noteworthy here is the substantial disparities between individual regions.

**Table 1:** Collation of hospitals with donation potential, active hospitals, the number of real organ retrievals and donors per million inhabitants in every province in 2018 (2).

**Province**	**Number of** **hospitals with** **donation potential**	**Number of active** **hospitals (2018)**	**Percentage of** **active hospitals (%)**	**Number of** **real donors**	**Number of donors** **per million inhabitants** **of province**
Dolnoślaskie	24	9	37.5	38	13.1
Kujawsko- Pomorskie	19	10	52.6	30	14.4
Lubelskie	22	7	31.8	19	9.0
Lubuskie	13	3	23.0	12	11.8
Łódzkie	27	3	11.1	14	5.7
Małopolskie	30	11	36.6	44	13.0
Mazowieckie	55	16	29.0	55	10.2
Opolskie	8	3	37.5	19	19.2
Podkarpackie	22	5	22.7	8	3.8
Podlaskie	15	5	33.3	14	11.8
Pomorskie	23	12	52.1	44	19.0
Slaskie	38	18	47.3	82	18.1
Swietokrzyskie	15	5	33.3	6	4.8
Warmińsko-Mazurskie	19	6	31.5	26	18.2
Wielkopolskie	38	19	50.0	55	15.9
Zachodniopomorskie	20	6	30.0	32	19.1
Summary	388	138	34.9	498	13.0

*(Modified by authors). Province, polish names of each voievodeship*.

**Figure 1:**
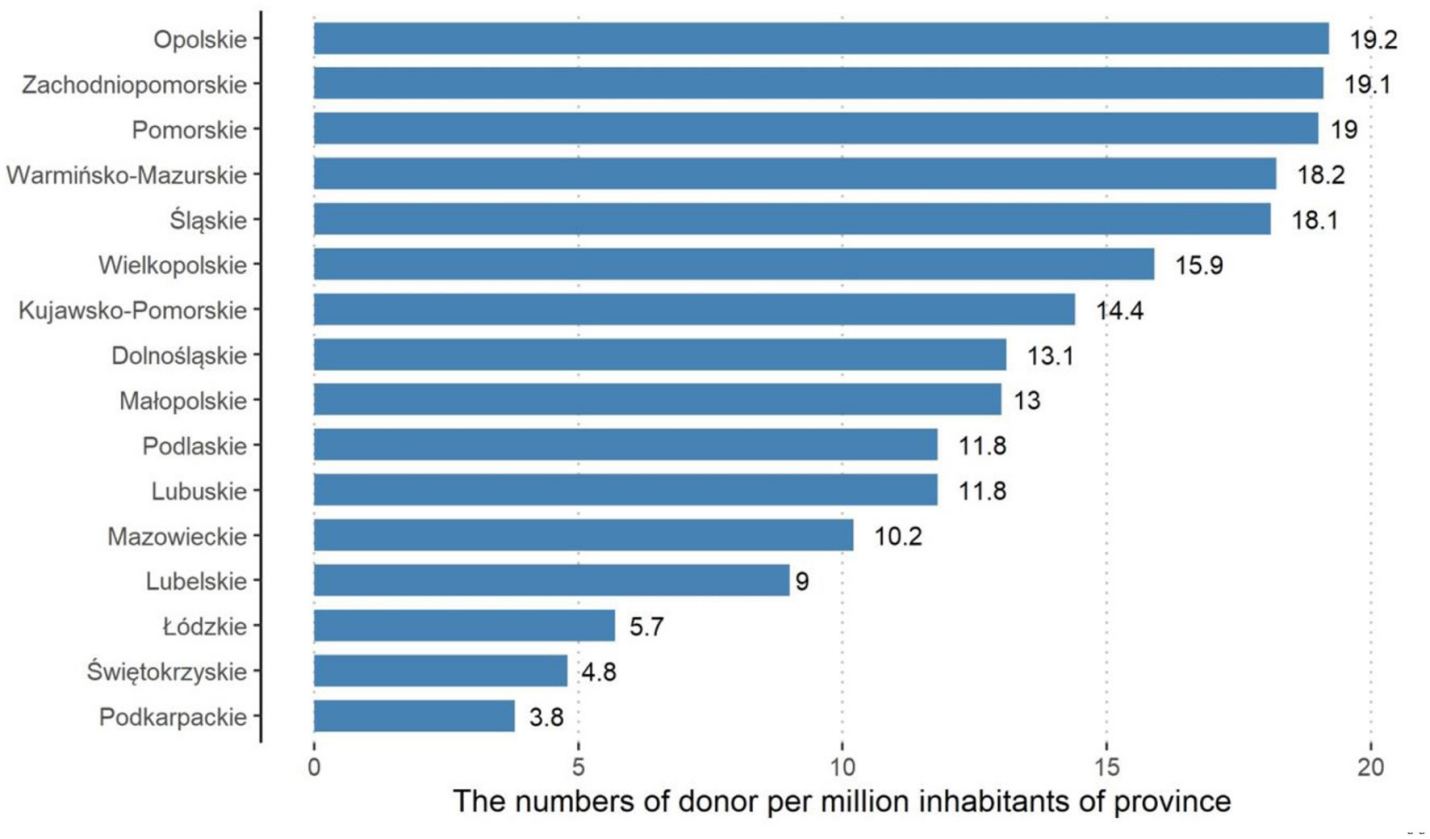
Numbers of donors per one million inhabitants. Province, polish names of each voievodeship (2).

**Figure 2:**
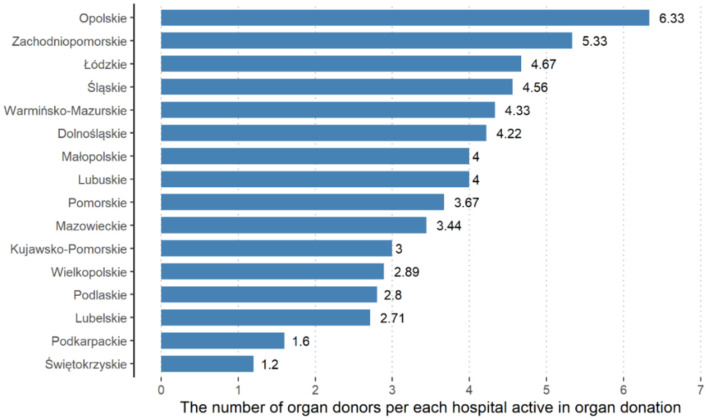
Numbers of donors in the facilities actively participating in organ transplantation per individual province. Province, polish names of each voievodeship (2).

The number of donor distributions (Table 1) per million inhabitants of particular provinces is presented in Figure 1. Figure 2 presents the number of donors per hospitals that actively participate in organ transplantation per individual province. In both cases, a small province of Opole was ranked first in Poland.

According to the data gathered by Poltransplant (2), the total number of patients qualified for an organ retrieval procedure in 2018 amounted to 638, 140 of whom were real donors, i.e., patients from whom organs were actually retrieved. In 140 cases (21.9%) physicians derogated from organ retrieval because of: a lack of authorization (79–12.4%) or medical indications (61–9.6%).

Brain vascular diseases (264–53%) and traumatic brain injuries (170–34%) were the main causes of brain death diagnosis and organ donor qualifications. Intoxication, hypoxia and mild brain tumors (60 cases−12%) were identified as the other causes.

Having analyzed the presented data, the authors concluded that there was still underutilized potential for organ donation in Polish hospitals. In order to identify possible obstacles in the diagnosis and organ donors qualification, the medical records covering the period of 2017–2018 were retrospectively analyzed. The data were acquired from the Anesthesiology and Intensive Care Department in lubuskie province, from one of 3 hospitals actively involved in qualification of organ donors in that region. The hospital is a university facility with 10 intensive care beds, 770 hospital beds and approximately 500 patients treated per year in an intensive care unit (ICU). The hospital is also a regional trauma center with a department of neurosurgery and neurotraumatology, an orthopedic-trauma unit and an emergency department. Taking into consideration a diverse profile of the hospital, the donation potential could be expected to be much higher, given a slight improvement of protocols and a meticulous identification of potential donors.

## Materials and Methods

The records of every patient admission in the analyzed period were reviewed and, consequently, two groups of patients were selected:

1. Patients admitted due to central nervous system (CNS) disabilities: brain vascular diseases and traumatic brain injuries resulting in: cerebral edemas, brain contusions, hypoperfusion of brain tissue, epidural hematomas, subarachnoid hematomas, intracranial hemorrhages, subarachnoid hemorrhages, diffuse axonal injuries–DBD (Donation after Brain Death) group (3).
2. Patients admitted after cardiac arrest with general, severe hypoxemic CNS injuries- DCD (Donation after Cardiac Death) group (3).

The following demographic factors of individual patients in each group were analyzed: gender, age, type of CNS injury, the criteria of suspected brain death, the length of ICU stay and treatment results.

The patients who potentially fulfilled brain death criteria but did not undergo the established qualification procedure were identified and then analyzed in detail. The aim was to determine why the qualification procedure was discontinued. These cases provided the grounds to construct three clinical scenarios in order to discuss them further herein.

### Description of the Statistical Analysis

The quantitative variables highlighted in this article were characterized in various cross-sections by basic statistical indicators like arithmetic mean, standard deviation and quartiles. Additionally, the distribution of these variables was represented graphically with box plots. The qualitative variables were summarized by numbers and percentages.

The comparison of the proportions of patients' responses to external stimuli in the neurological group of patients and in the group of patients with cardiac arrest was tested using the chi-square test for proportions.

An analysis of the correlation was performed to measure connections and strengths of dependence between the selected variables that characterized the clinical state of a patient. In the case of quantitative variables, a non-parametric Spearman's correlation coefficient (r_s_) was calculated. The influence of the qualitative on the quantitative variable was verified based on the statistical significance of ANOVA model. The Pearson correlation coefficient (r) in this case measured the strength of the linear relationship between the observed measurements of the quantitative feature and their values resulting from the predictions of this model.

For all the statistical tests the level of statistical significance was defined as 0.05. The analyses were conducted using R program (4).

## Results

The results of 229 patients hospitalized in Intensive Care Unit from 2017 to 2018 were included into the analysis. The patients were divided into 2 subgroups; vascular or traumatic cerebral injury group (156 patients, *n* = 65,5%) and post-cardiac arrest group (73 patients, *n* = 34,5%). In both groups further selection was made focusing on particular features: no signs of reactions to external stimuli, suspicion of brain death, angio-computed tomography (CT) and organ donation performed (Table 2).

**Table 2:** The number of patients in two subgroups in relation to suspicion of brain death, angio CT performed and actual organ donation.

	**Number (%) of patients hospitalized in Intensive Care Unit during 2017-2018 included in the analysis** ***N*** **=** **229 (100%)**	
	**DBD group** ***N* = 156 (68%)**	**DCD group** **(suspected hypoxic-ischemic** **encephalopathy) *N* = 73 (32%)**
Suspicion of brain death	39 (17%)	14 (6%)
Angio-CT performed	15 (6%)	2 (1%)
Organ donation	9 (4%)	1 (0.4%)

*N, number of patients in each group*.

### The Group of Patients After Cardiac Arrest (DCD Group)

Cardiac arrest with effective resuscitation was the reason for ICU admission in 73 (34.5%) patients during the analyzed period. In out-of-hospital resuscitation procedures, defibrillation rhythm was observed in 25 patients (34.25% of the analyzed group) and non-defibrillation rhythm was recorded in 48 (65.75%). Women and men constituted 28 (38.36%) and 45 (61.64%) of the group, respectively. The average age was 65 years and the mean length of ICU stay was 5.5 days. Each patient of this group demonstrated features of CNS injuries in the course of post-resuscitation syndrome shown as hypoxic-ischemic encephalopathy of different severity (ranging from mild alterations to the suspicion of brain death). 27 (37%) patients did not present any reactions to external stimuli–Glasgow Coma Scale (GCS) 3. Suspected brain death occurred in 14 (19.18%) patients and angio-CT was performed in 2 of them (2.74%). Eventually, organ retrieval was performed in one case (1.37%). Thirty four patients (53.42%) died in the ICU, and 39 patients (46.58%) were relocated to other wards (as a result of an improvement or palliative care administration).

In 19 patients (26%), a short hospital stay (<2 days) and a quick death due to deep hemodynamic instability as a result of the primary causes of cardiac arrest, did not allow for the implementation of the procedure to declare brain death. Four patients (5.48%) had comorbidities disqualifying them as potential donors, and 3 patients (4.11%) were chosen for further analysis.

### The Group of Patients With Vascular or Traumatic Cerebral Injuries (DBD Group)

**Figure 3:**
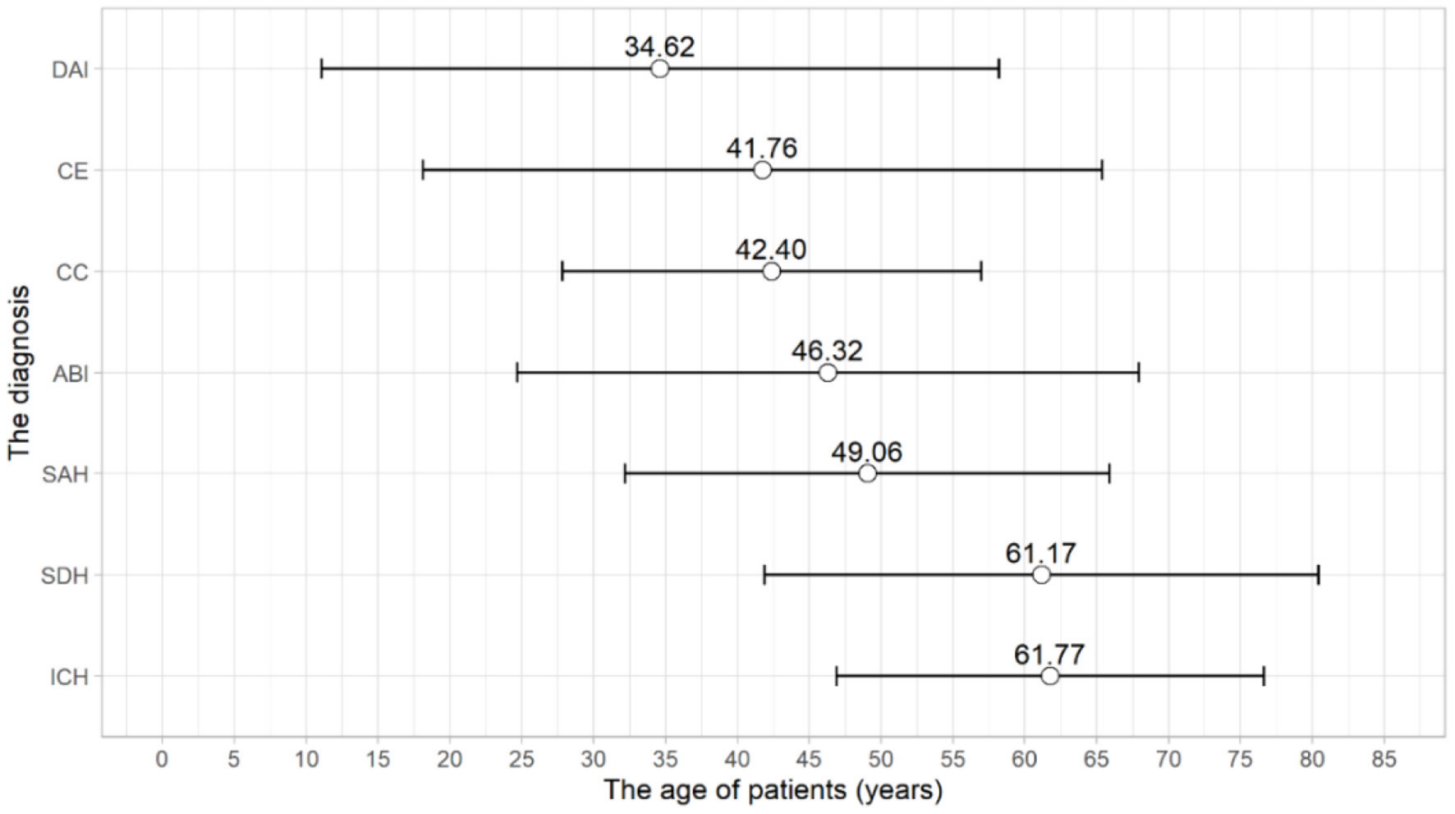
The distribution of mean age and the standard deviation of a patient's age depending on CNS injury cause in DBD group. CNS, Central Nervous System.

After the analysis of medical records, 156 patients were qualified for this group. The number of hospitalized women and men amounted to 54 (34.62%) and 102 (65.38%), respectively. The mean age of the patients was 53. In this group, a clear correlation between a patient's age and the cause of CNS injury was detected (Figure 3). The mean ICU length of stay was found to be increased as compared to the cardiac arrest group to approximately 7 days. There were 90 patients (57.69%) relocated to other wards, and 66 (42.31%) of them died during ICU stay.

The types of brain injury are described in Table 3.

**Table 3:** Types of cerebral injury in the analyzed DBD group.

**Type of** **CNS injury**	**Number of cases** **(*n*)**	**Percentage** **distribution (%)**	**Brain death** **suspicion**	**Number of** **organ retrievals**
SDH	36	23.08	9	2
SAH	32	20.51	12	5
CE	29	18.59	7	0
ICH	22	14.10	6	0
ABI	19	12.18	3	1
CC	10	6.41	1	0
DAI	8	5.13	1	0

*SDH, Subdural Hemorrhage; SAH, Subarachnoid Hemorrhage; CE, Cerebral Edema; ICH, Intracerebral Hemorrhage; ABI, Anoxic brain Injury; CC, Contusio Cerebri; DAI, Diffuse Axonal Injury*.

In this group of patients, no reaction to any stimuli (GCS 3) was observed in 43 (27.56%) patients during their ICU stay. The suspicion of brain death was recorded in 39 (25%) patients. Angio-CT of cerebral vessels was performed in 15 (9.69%) patients and the declaration of brain death and organ donation was made for 8 (5.13%) hospitalized patients.

**Figure 4:**
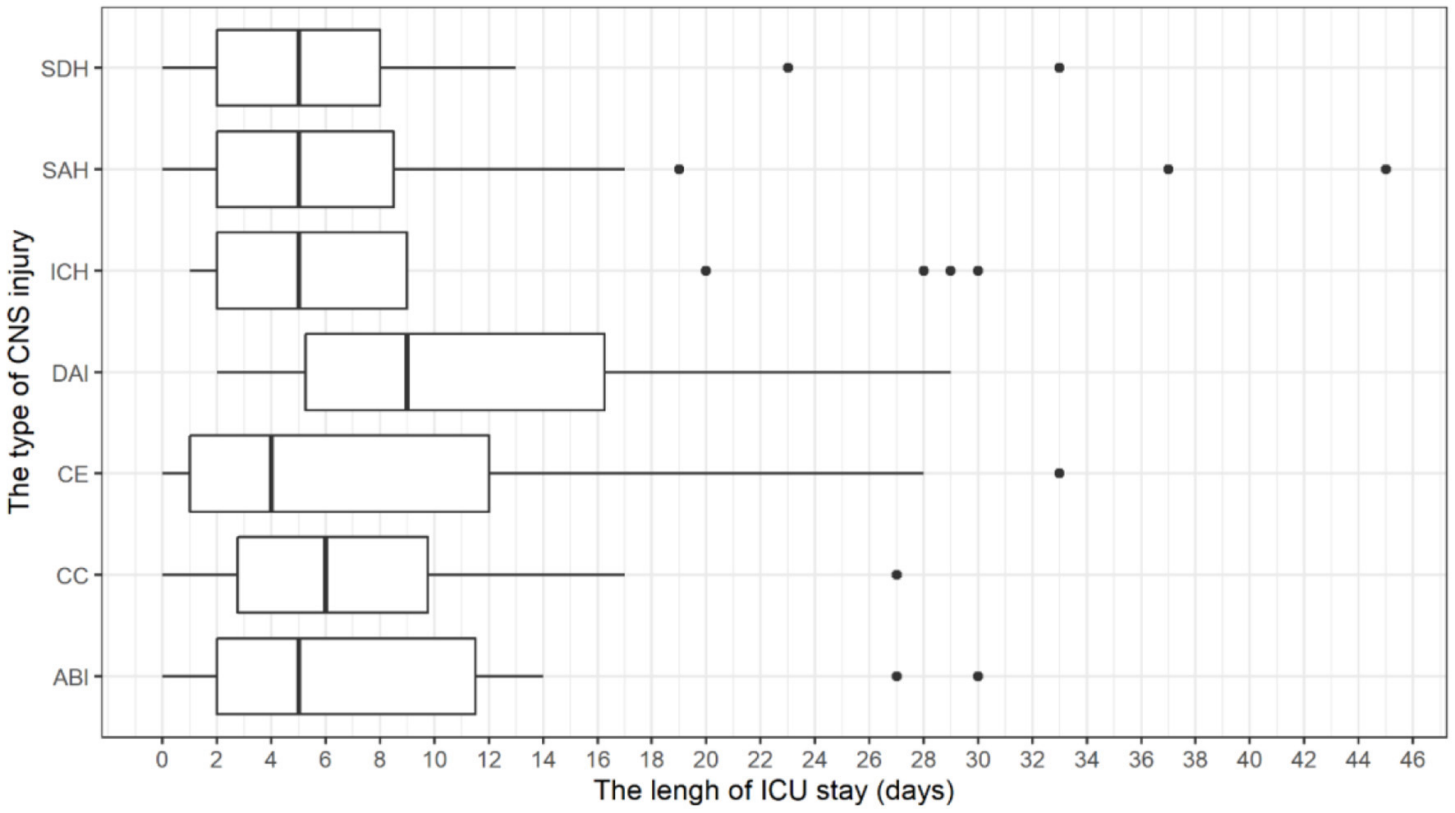
Distribution of ICU length of stay with regard to the type of CNS injury in the DBD group. CNS, Central Nervous System.

In 43 patients who potentially qualified for being declared brain dead, 7 (4.49%) were disqualified because of the existing comorbidities, 14 were excluded due to a short hospital stay (Figure 4) (fast relocation to other hospital wards or death in <2 days of ICU stay) and 13 cases were chosen for further analysis for the reasons which eventually led to an unfinished procedure of brain death declaration and did not allow for the completion of organ retrieval.

The group of patients with traumatic subdural hematomas was dominated by men of mean age of 45–55 years and a hospital stay no longer than 8 days. Brain death was suspected in 9 (25%) out of 36 patients in this group and 2 organ retrievals were performed.

In the group of 32 study patients (20.51% of all the patients) with subarachnoid hemorrhages, brain death was suspected in 12 of them, and, after declaring brain death, organ retrieval was performed in 5 patients, which made them the most numerous group of organ donors (Table 3).

### Comparison of the DBD and DCD Group of Patients

**Figure 5:**
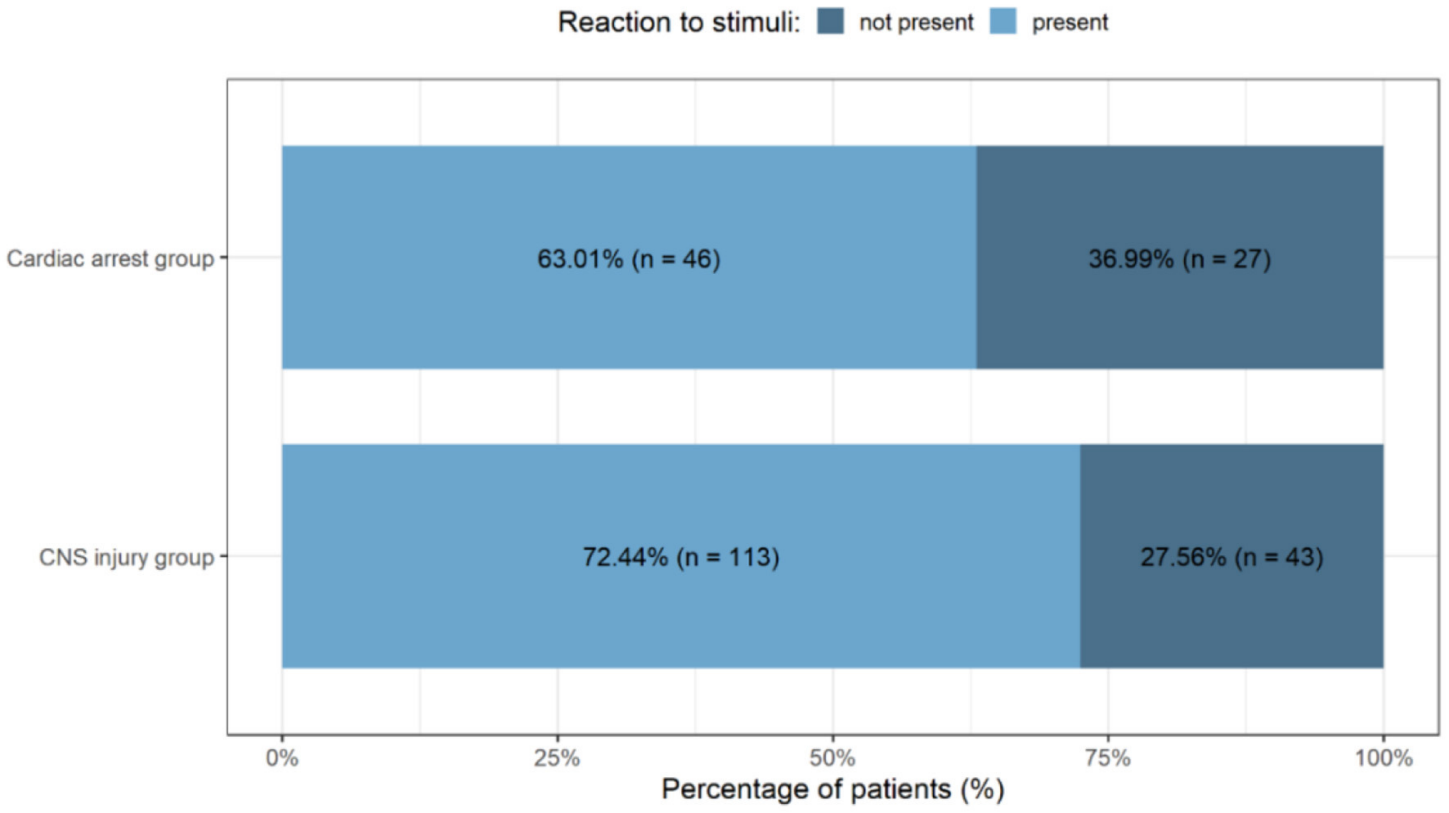
Presence of a reaction to external stimuli in the neurological group of patients and in the cardiac arrest (DCD) group. CNS, Central Nervous System.

No reaction to external stimuli (after exclusion of sedation), GCS 3, was the main factor suggesting a potential brain death. In the group of patients with cardiac arrest, this was a more frequent symptom, recorded in 27 cases i.e., 37% of all the cardiac arrest group of patients, when compared to the neurological injury group where the symptom was identified in 27.56% (Figure 5). However, the percentage of patients non-responsive to external stimuli did not differ statistically significantly in these two groups (χ^2^ = 1.66, *p*-value = 0.1976).

In the group of patients with neurological causes of suspected brain death and no reaction to external stimuli, angio-CT imaging was performed in 15 out of 43 cases in order to either confirm or exclude that suspicion. In the cardiac arrest group such an examination was done in 2 cases.

### The Group of Patients Selected for Further Detailed Analysis Medical History

For further analysis, 3 cases were selected from the cardiac arrest group and 13 cases from the group with neurological causes of brain injury, in which the procedure of declaring brain death was not performed, despite fulfilling all the essential clinical criteria.

CNS injuries in the DBD group were caused by subarachnoid hemorrhages in 3 patients, cerebral edemas in 3 patients, subdural and intracerebral hematomas in 6 cases and anoxic brain injury in one patient. All patients had low GCS scores (3–5 points), age differences were notable (3–77 years old), each of them had a CT performed on the day of ICU admission, a control CT examination was done in 10 cases, usually 48 h after finishing sedation, and no neurological improvement was registered. Angio-CT was performed only in 3 cases.

Fifteen patients died in the ICU (2nd-14th day), and one of them was discharged for the purpose of relocation. Sedation was implemented in 8 patients with an infusion of propofol and fentanyl, one patient received thiopental and in 2 cases midazolam and fen-tanyl were applied. Five patients did not receive any sedation (GCS 3). The duration of se-dation was different for each individual (1-2-3-4-6-13 days). One patient received intra-cranial pressure (ICP) monitoring.

During the analysis, a correlation of factors that had an impact on the decision to implement the procedure of declaring brain death was noticed. The brain death suspicion was found to be based mainly on the outcome of physical examination and assessment by GCS. GCS score at a minimal level (3 points) combined with the shortest duration of sedation was the main reason for performing a brain CT scan. The strongest correlation was identified in the cases where brain imaging computed tomography was combined with the angiographic option- *angio* (r = 0.69, *p* = 0.0032). A lack of blood perfusion in the angio-CT led directly to the implementation of an instrumental examination to confirm brain death. This type of tomographic examination was performed in 4 analyzed cases. The length of stay in the ICU correlated positively with the time of the sedation period (r_S_ = 0.43, *p* = 0.0469) and the decision to perform brain imaging (r_S_ = 0.58, *p* = 0.0087), but the observed correlation with the declaration of brain death was not statistically significant (*r* = 0.30, *p* = 0.266).

## Discussion

The number of declared brain deaths is the major factor that impacts the number of organ retrievals for transplantation. It is assumed that in the countries which perform more than 20 transplants per 1 million inhabitants, brain death is declared in over 40 cases per million (5). These countries include Spain, Portugal, Puerto Rico, Croatia, USA, France, Austria, Belgium and Italy and they maintain a constantly high number of organ retrievals, regardless of technological and scientific development regarding all causes of irreversible brain injury (6). Such results are affected by a range of factors including care plans for terminally ill patients, the palliative treatment of critically ill patients in ICUs, early planning of treatment strategies and prognosis assessments of patients with central nervous system injuries (5, 7).

A rise in the number of deceased organ donors is dependent on the increasing number of brain death diagnoses. Monitoring the relation between the quantity of potential organ donors to the real number of organ retrievals is an efficacy criterion for the whole health care system in the field of intensive care and transplantology. Swiss data (8) showed a conversion factor i.e., the number of real donors as a percentage of potential donors, at the level of 46.7%. The conversion factor in the analyzed ICU of this study was found to equal 17%.

Another assessment criterion is the estimate of the donor potential, which is the ratio of patients declared brain dead to the number of ICU beds per 1 year (9–11). The lowest value should be equal to 1 declared brain death/ 1 ICU bed/ 1 year. In 2017–2018, there should have been 20 patients with declared brain death in the analyzed ICU whereas brain death diagnoses and organ retrievals were performed in only 9 patients, so the donation factor was 45%.

In the scientific literature concerning constraints of organ donation in patients with diagnosed brain death (12, 13), several main factors can be found, such as inadequate hospital or ICU funding systems, a lack of health care professionals, a poor identification of the staff with their workplace, different employment forms, reluctance to make difficult choices, fear of failure in contacts with the patient's family. All those reasons are observed and noted in most of the health care systems (14).

Many government institutions offer training programs for hospital facilities and personnel regarding protocols and care for donors. Some emphasis is also placed on the promotion of new local organizational solutions. This issue is illustrated by our third clinical case in Results section (Third clinical case.) when witnessing a death of a child must have been a huge emotional burden on medical personnel.

In the performed analysis, a few essential elements of medical procedures and organization at ICUs were identified as the factors which may have an impact on the quantity of potential and real donors. The identification of patients with low GCS score (3–4 points) who are not treated with deep sedation is a basic feature that draws attention during routine procedures performed at ICUs (15). Performed at least twice a day, GCS assessments and neurological examinations to check cranial nerves reflexes and the presence of apnea aim at identifying the patients who are undergoing the process of brain death.

Other features that may direct the physicians' attention to a potential brain death include sudden hyperdynamic disturbances of the circulatory system (vegetative storm) or hypodynamic disturbances (vasoplegia) which are often associated with hypothermia (16, 17). The presence of these circulatory alterations is dependent on the brain injury etiology, its duration and any therapeutic procedures performed. This problem is clearly described herein as our second clinical case in Results section (Second clinical case). We identified a 24-h delay in the performance of necessary imaging after a vegetative storm was observed. Eventually, the undue delay resulted in a failure to finish the procedure of brain death declaration.

Our analysis of the patient population showed a relationship between the ICU length of stay and a suspicion and diagnosis of brain death. In many cases, the length of a patient's stay was insufficiently long (<48 h) to complete the procedure of brain death declaration. This, in turn, was associated with a shortage of ICU beds and the necessity to relocate patients who showed no indications for advanced treatment implementation. Several of them met the preliminary criteria of brain death suspicion (low GCS, CNS injury etiology). After the patient transfer to ICU of lower referral, however, brain death declaration procedures were not continued due to organizational issues. This problem clearly concerns our first clinical case in Results section (First clinical case). The patient who could have been a potential organ donor was transferred to the hospital of lower reference and never became one.

The duration of sedation was found to be correlated directly with low GCS score and the brain death declaration procedure. Currently available clinical data (18) support the procedure of adjusting the depth and type of sedation to CNS damage etiology and overall clinical situation (the presence of mechanical ventilation, hemodynamic and metabolic stability). The depth of sedation should be monitored with Richmond Agitation- Sedation Score (RASS) (19) and other methods like Confusion Assessment Method for the ICU (CAM-ICU) (20). Adjusting sedation to the circadian rhythm and a regular decrease of the dose to perform adequate neurological assessments and to determine other parameters to describe brain homeostasis are essential for the objective evaluation of cerebral functions, as well as diagnosis of brain death.

Propofol is the most popular sedative which is commonly used in intensive care units. The initiation of a clinical diagnosis of brain death is recommended only with propofol serum concentrations of <0.4 μg / ml (21).

Monitoring brain functions in critically ill patients with primary or secondary CNS injuries is considered as a prognostic factor inextricably linked to the suspicion of brain death. Intra Cranial Pressure monitoring (ICP) and Cerebral Perfusion Pressure monitoring (CPP) enable the measurements of cerebral vascular perfusion (22). ICP and CPP monitoring in patients with potentially lethal brain injuries can accelerate the diagnosis and lead to the brain death declaration procedure before irreversible systemic disturbances occur.

New technologies in monitoring CNS functions facilitate the identification of brain death and the procedure implementation in accordance with to current legislative regulations. The advantages of these methods include their non-invasiveness, easy implementation and continuity: the features that seem to be of great importance in the face of a typical shortage of medical staff in ICUs. One of them is Near Infrared Spectroscopy (NIRS) which assesses the numerical ratio of oxygenated to non-oxygenated hemoglobin [delta (HBO2)]/[delta(HB)] in cranial tissues (skin, bones, muscles, neural tissue) (23). A modification of the standardized protocol might be needed to assess NIRS depending on expiratory oxygen concentrations (fiO2 0,2-0,5-1,0). Brain dead patients had a ratio [delta (HBO2)] /[delta(HB)] recorded over 1.4-1.5 which indirectly (and non-invasively) proves cerebral perfusion disturbances. Further clinical investigation in this field may be promising; it is a low-cost, non-invasive and easy method of the identification of potential donors in intensive care units.

Similarly, the use of microdialysis–an analysis of proteins and other substances or metabolites in damaged/dying brains (lactate/pyruvate ratio: L/P > 30) (24) can be an interesting alternative to current technologies.

A brain CT scan was a key element of diagnostic examination for patients in the neurological group and in the cardiac arrest group in our study population. In all of 16 cases chosen for further analysis, a standard head CT was performed in the first 24 h, usually before admission to the ICU. The subsequent examination was scheduled individually, depending on clinical contexts. Angio-CT was performed in one case prior to the time of ICU admission, in 3 cases during ICU stay (2^nd^-8^th^-14^th^ days). The main indication for CT was no improvement observed in the patient's neurological state despite the termination of sedation. The period between termination of sedation and the CT examination was 48 h. Angio-CT was not performed in the neurological group of patients who suffered from subarachnoid hemorrhages (SAH). In one case, angio-CT was done during the diagnostic process before ICU admission and no signs of brain perfusion were identified. Despite that, the patient received sedation for 5 days of hospitalization and was not qualified as a potential organ donor. It is hard to say why the CT scan in *angio* option was not performed–it may have happened due to the cost restriction. In some cases, it was an independent decision of the physician on duty.

Angio-CT performed on ICU admission may facilitate earlier identification of potential organ donors, as a result of the high diagnostic specificity for brain death (25). This, in turn, would trigger faster implementation of brain death protocols leading to an increased pool of organs available for transplantation.

There is a lot of data (26) supporting the whole body angio-CT as a method of assessment of organ perfusion in different vascular compartments which enables fast evaluation of cerebral, lung, heart and abdominal organs anomalies. Contrast nephrotoxicity may be considered in these circumstances, however, none of our patients had a contrast induced injury (25–28). Technological development of superfast CT scans and software advancement can reduce the requirements for contrast dosing.

## Conclusions

An increase in organ retrievals for transplantation depends on several socio-economic factors like health care financing, social acceptance of organ donation and retrieval consent. Local diagnostic and therapeutic protocols in the potential organ donor group is not of lesser importance. The etiology and medical history of cerebral injuries may suggest the risk of brain death. The factors that determine the identification of real donors and the implementation of procedures to declare brain death with organ retrievals include a decrease in sedation depth, monitoring of cerebral hemodynamic and metabolic status, frequent neurological examinations with GCS assessments and angio-CT done at early stages of proceedings.

## Data Availability Statement

The raw data supporting the conclusions of this article will be made available by the authors, without undue reservation.

## Ethics Statement

Ethical review and approval was not required for the study on human participants in accordance with the local legislation and institutional requirements. Written informed consent from the [patients/participants OR patients/participants legal guardian/next of kin] was not required to participate in this study in accordance with the national legislation and the institutional requirements.

## Author Contributions

Conceptualization and writing—original draft preparation: BK. Methodology: MT. Validation: MM, BK, and MT. Formal analysis: MW and GZ. Resources: OF and JK. Data curation: AB and MT. Writing—review and editing and supervision: MM. Visualization: JK. All authors have read and agreed to the published version of the manuscript.

## Funding

This work was supported by funds from the University of Zielona Gora (No. 222267/E-545/S/2021).

## Conflict of Interest

The authors declare that the research was conducted in the absence of any commercial or financial relationships that could be construed as a potential conflict of interest.

## Publisher's Note

All claims expressed in this article are solely those of the authors and do not necessarily represent those of their affiliated organizations, or those of the publisher, the editors and the reviewers. Any product that may be evaluated in this article, or claim that may be made by its manufacturer, is not guaranteed or endorsed by the publisher.
